# Formation and Entrapment of Tris(8-hydroxyquinoline)aluminum from 8-Hydroxyquinoline in Anodic Porous Alumina

**DOI:** 10.3390/ma9090715

**Published:** 2016-08-24

**Authors:** Shohei Yamaguchi, Kazunori Matsui

**Affiliations:** Department of Applied Materials and Life Science, Graduate School of Engineering, Kanto Gakuin University, 1-50-1 Mutsuurahigashi, Kanazawa-ku, Yokohama, Kanagawa 236-8501, Japan

**Keywords:** anodic porous alumina, tris(8-hydroxyquinoline)aluminum, 8-hydroxyquinoline, absorption spectra, fluorescence spectra

## Abstract

The formation and entrapment of tris(8-hydroxyquinoline)aluminum (Alq_3_) molecules on the surface of anodic porous alumina (APA) immersed in an ethanol solution of 8-hydroxyquinoline (HQ) were investigated by absorption, fluorescence, and Raman spectroscopies. The effects of the selected APA preparation conditions (galvanostatic or potentiostatic anodization method, anodizing current and voltage values, one- or two-step anodizing process, and sulfuric acid electrolyte concentration) on the adsorption and desorption of Alq_3_ species were examined. Among the listed parameters, sulfuric acid concentration was the most important factor in determining the Alq_3_ adsorption characteristics. The Alq_3_ content measured after desorption under galvanostatic conditions was 2.5 times larger than that obtained under potentiostatic ones, regardless of the adsorbed quantities. The obtained results suggest the existence of at least two types of adsorption sites on the APA surface characterized by different magnitudes of the Alq_3_ bonding strength. The related fluorescence spectra contained two peaks at wavelengths of 480 and 505 nm, which could be attributed to isolated Alq_3_ species inside nanovoids and aggregated Alq_3_ clusters in the pores of APA, respectively. The former species were attached to the adsorption sites with higher binding energies, whereas the latter ones were bound to the APA surface more weakly. Similar results were obtained for the Alq_3_ species formed from the HQ solution, which quantitatively exceeded the number of the Alq_3_ species adsorbed from the Alq_3_ solution. Alq_3_ molecules were formed in the HQ solution during the reaction of HQ molecules with the Al^3+^ ions in the oxide dissolution zone near the oxide/electrolyte interface through the cracks and the Al^3+^ ions adsorbed on surface of pore and cracks. In addition, it was suggested that HQ molecules could penetrate the nanovoids more easily than Alq_3_ species because of their smaller sizes, which resulted in higher magnitudes of the adsorption.

## 1. Introduction

Anodic porous alumina (APA) has been used in various industrial applications because of the excellent corrosion and abrasion resistance properties, while its high porosity makes it suitable for decorative coloration techniques such as electroplating, painting, and dyeing [1,2,3]. Recently, APA processing has attracted remarkable interest in various scientific and technological fields such as electrochemistry, material science and, especially, nanotechnology, since the pioneering work on self-ordered nanoporous alumina conducted by Masuda et al. [4]. The usage of ordered pore array structures as a template allows the preparation of a large variety of nanomaterials and nanocomposites for various applications, including photonic crystals, light-emitting devices, capacitors, biosensors and medical equipment [3,5,6,7,8,9,10,11,12].

**Figure 1:**
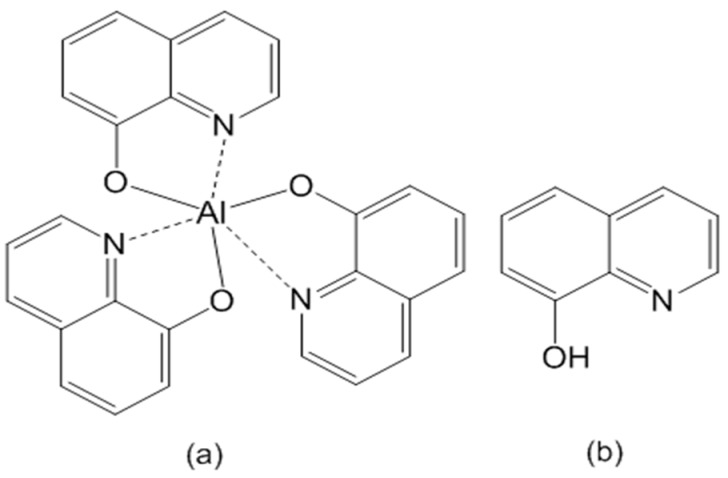
Molecular structures of (**a**) Alq_3_ and (**b**) HQ species.

Tris(8-hydroxyquinoline)aluminum (Alq_3_, Figure 1a) is known as a fluorescent complex showing electroluminescence with a high quantum yield [13]. Therefore, Alq_3_ embedded in APA has attracted significant interest in connection with optical applications [8,9,10,14,15,16]. Xu et al. [14,15] reported that the photoluminescence spectra of the APA samples with pores of about 10–15 nm immersed in a 0.01 M chloroform solution of Alq_3_ for 21 h exhibited a blueshift from 520 nm to 488 nm, whereas a smaller blue shift from 518 nm to 510 nm was observed for the treated APA samples with the pore sizes of about 40–50 nm owing to the suppression of Alq_3_ aggregation inside the narrower nanometer-sized pores. In contrast, Huang et al. [9] suggested that such blueshifts could be attributed to the confinement of Alq_3_ molecules inside nanovoids of the nanopore walls, because similar blueshifts from 516 nm corresponding to a solid Alq_3_ film to 498 nm for Alq_3_ were detected for the Alq_3_ species embedded in APA structures with nanopore sizes ranging from 23 to 45 nm. The authors concluded that Alq_3_ molecules could diffuse into the nanovoids with sizes of 1–2 nm through the cracks connected to the pores surface [17,18]. Mohammadpour et al. [16] prepared two APA samples containing cylindrical nanopores with diameter around 20 nm; one sample was immersed in a 1 mM solution of Alq_3_ in chloroform for 21 h followed by thorough rinsing with pure chloroform, while the other sample was dipped and dried several times in a 10 mM solution of Alq_3_. The former sample exhibited a fluorescence peak at 498 nm, and the second one—a peak at 510 nm. According to the obtained steady-state and time-resolved data, the former peak could be attributed to the Alq_3_ molecules dispersedly embedded in the APA nanovoids with sizes of 1–2 nm, while the latter one corresponded to the Alq_3_ aggregates adsorbed on the APA pore surface. However, reaching consensus appears to be very difficult because these samples were prepared under different anodic and embedment conditions. Moreover, the assignment of fluorescence peaks must be conducted carefully because of the photoluminescence of the APA substrate [9,19,20,21]. Hence, the details of the mechanism describing Alq_3_ embedment in the APA matrix should be studied more thoroughly. 

The adsorption studies of various dyes on the APA surface revealed that the adsorbed amount of Alq_3_ in an aqueous solution of negatively-charged sodium dodecyl sulfate is three times larger dissolving Alq_3_ than that in *N*,*N*-dimethylformamide dissolving Alq_3_, while no pyrene adsorption was observed when an APA substrate was immersed in a pyrene-containing *N*,*N*-dimethylformamide [22]. Despite the relatively small amount, a fraction of Alq_3_ remained on the APA surface. It is generally accepted that dye adsorption on the APA pore surface is mainly caused by the electrostatic interaction between the positively-charged APA surface and the negatively-charged dye molecules. Thus, the following questions may be asked: what kind of interaction is this? What are the locations of the trapping sites inside the pores?

In our previous study, Alq_3_ species were entrapped in APA by immersing it in a solution containing 8-hydroxyquinoline (HQ, Figure 1b) [23]. As a result, the HQ molecules reacted with the APA surface, leading to the formation of APA-embedded Alq_3_ species (the APA support was prepared via one-step anodization at constant current conditions, which represent a commonly used industrial dyeing method). The detailed reaction mechanism remained unclear, because the utilized APA surface did not show clear nanoporous structure. Afterwards, we investigated the formation of Alq_3_ from HQ on the APA surface characterized by self-ordered nanopores, which was prepared by two-step anodization at a constant voltage [24]. The obtained results were similar, although the detailed mechanisms of Alq_3_ formation from HQ-containing solution and its subsequent entrapment were not very clear either. In this work, we investigated the formation of Alq_3_ species from HQ-containing solution on the surface of APA prepared in sulfuric acid under various anodizing conditions, as well as their adsorption and desorption behavior. The obtained results can be potentially utilized for novel optical materials and surface modification techniques. 

## 2. Results

### 2.1. SEM Analysis of APA Surfaces

**Figure 2:**
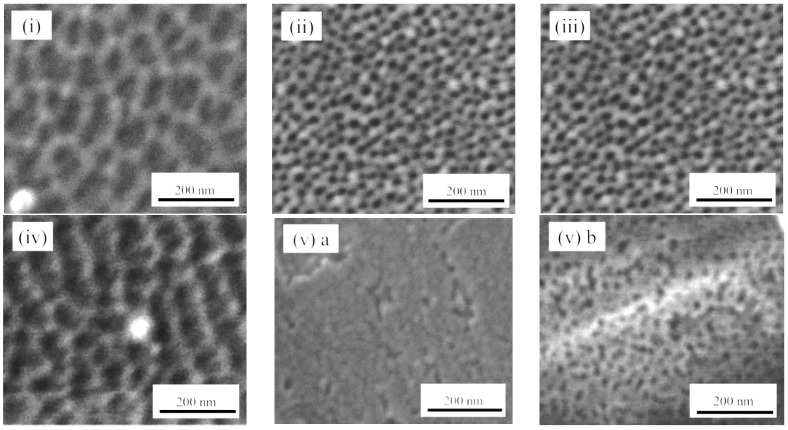
SEM images of the APA surfaces prepared in sulfuric acid under the anodizing conditions listed in Table 1. Electropolishing pretreatment of the Al surface: (**i**) one-step, 20 V in 0.3 M; (**ii**) two-step, 20 V in 0.3 M; (**iii**) two-step, 10 V in 0.3 M; (**iv**) one-step, 3 A/dm^2^ in 1.5 M. Degreasing pretreatment of the Al surface: (**(v)a**) one-step, 3 A/dm^2^ in 1.5 M as-prepared and (**(v)b**) subsequently electropolished APA surfaces.

Figure 2 shows the scanning electron microscope (SEM) images of the APA surfaces anodized under various conditions listed in Table 1. The utilized preparation conditions were selected to examine the effects of the anodizing and pretreatment procedures on the Alq_3_ entrapment and reactivity of HQ species with the APA. As shown in Figure 2(i) and (ii), the surface prepared via two-step anodization at a constant voltage of 20 V (ii) is characterized by a significantly more ordered structure as compared to the surface obtained by one-step anodization (i), which exhibits non-circular pores with sizes ranging from 20 to 70 nm. The average pore diameter (*D_p_*) in Figure 2(i) was equal to approximately 47 nm, whereas *D_p_* of the pores displayed in Figure 2(ii) was 33 nm. Figure 2(iii) shows the APA surface via two-step anodization at a constant voltage of 10 V. The resulting pore size (18 nm) was about a half of the value obtained at a voltage of 20 V (ii), which was in good agreement with the data reported in previous studies [1,3].

**Figure 3:**
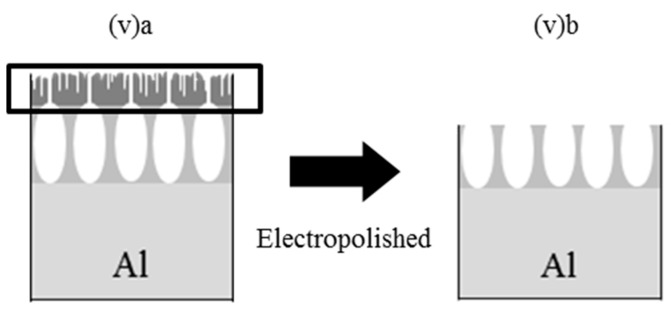
A schematic diagram of the APA preparation under conditions (v): (**a**) as-grown surface and (**b**) the sample obtained after removing the surface oxide layer via electropolishing.

Figure 2(iv) and (v)a reveal the difference in surface morphology observed after the electropolishing and degreasing steps, respectively, for the APA samples obtained via one-step anodization in a 1.5 M sulfuric acid at a constant current density. The resulting SEM image contains disordered pores, and the average magnitude of *D_p_* from the structures depicted in Figure 2(iv) was very similar to those of Figure 2(i). On the other hand, Figure 2(v)a shows no observable pores, but a rough surface with irregularly-shaped holes and cracks for degreased surface. In order to elucidate this difference, the surface of the sample depicted in Figure 2(v)a was further electropolished, leading to the appearance of pores (Figure 2(v)b). The obtained results indicate that the surface state results from the incomplete anodization accompanied by the formation of a with the cracked and pin-holed oxide layer [3]; anodization leads to form embryo of pores under the oxide layer due to the penetration of electrolyte mostly through the local cracking and imperfections. In other words, the APA pore structure resembles a bottle capped with the barrier oxide with a lot of small inlet channels. Hence, the removal of the surface oxide layer uncovered the hidden pores (see Figure 3); similar results were obtained for the APA surface prepared under the condition (vi). Therefore, it can be concluded that the elelctropolishing pretreatment removes the native oxide surface and accelerates pore formation, producing samples with different surface morphologies. 

The pore characteristics are summarized in Table 2: pore diameter (*D_p_*), cell diameter (*D_c_*), oxide film thickness (*t*), pore density (*n*), and inner pore surface area per 1 cm^2^ of APA surface (*S_p_*), which was estimated assuming the cylindrical pore shape. The values of *n* and *S_p_* were calculated according to the Equations (1) [25] and (2) listed below and were consistent with the values previously reported [1]:

(1) $$n=1\times 10^{14}/(\sqrt{3}\times {D_{c}}^{2})$$

*S_p_* = *n*π(10^3^*D_p_* × *t* + (*D_p_*^2^/4) (2)

### 2.2. Spectroscopic Characterization of the Alq_3_- and HQ-Species Adsorbed on the APA Samples Prepared under the Galvanostatic Method via One-Step Anodization of Degreased Al 

**Figure 4:**
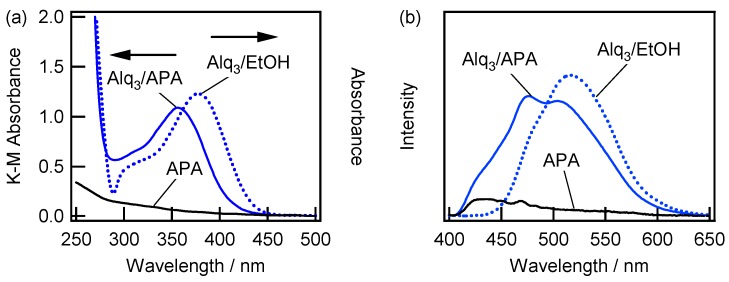
(**a**) Absorption and (**b**) fluorescence spectra obtained for the Alq_3_/EtOH (1 mM) and Alq_3_/APA systems (pure APA was used as control). The APA was prepared under conditions (v). The excitation wavelength was 350 nm.

Figure 4a,b show the absorption and fluorescence spectra obtained for the Alq_3_-containing ethanol solution (Alq_3_/EtOH) and APA samples immersed in Alq_3_/EtOH (Alq_3_/APA), respectively, (the related spectra of pure APA were used as control, and the APA was prepared under condition (v)). The absorption spectrum for Alq_3_/EtOH contains a strong peak at a wavelength of around 255 nm (not shown here) and a relatively weak peak at 377 nm, which are in good agreement with the results reported in the previous study [26]. The absorption spectrum recorded for the Alq_3_/APA system exhibits peaks at 257 and 358 nm, which are similar to those obtained for Alq_3_ entrapped in a self-ordered nanoporous alumina membrane [14,15], indicating the adsorption of Alq_3_ species on the APA surface. The fluorescence spectrum for Alq_3_/APA (Figure 4b) shows a blue shift of the peak wavelength from 513 nm (observed for Alq_3_/EtOH) to 480–505 nm, as have been reported elsewhere [14,15]. The absorption spectrum for pure APA does not show clear peaks in the wavelength regions of 300–400 nm (Figure 4a), while its photoluminescence intensity was much smaller than those observed for Alq_3_/APA under the same observation conditions (Figure 4b). Hence, the obtained results suggest that the effect of the APA surface characteristics on both the absorption and fluorescence properties of the embedded Alq_3_/APA systems is negligibly small. It should also be noted that the obtained Alq_3_/APA fluorescence spectrum is characterized by the split peaks with the maxima at around 480 and 505 nm in contrast to the broad single peak obtained in previous studies [9,14,15,16].

**Figure 5:**
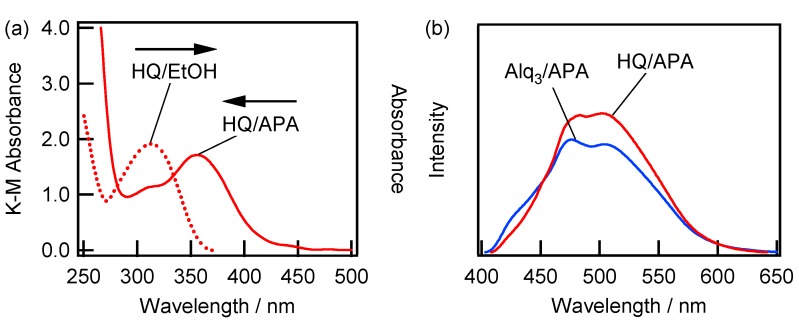
(**a**) Absorption spectra obtained for the HQ/EtOH (1 mM) and HQ/APA (v) systems; and (**b**) fluorescence spectra recorded for the Alq_3_/APA and HQ/APA (v) samples. The excitation wavelength was 350 nm.

Figure 5a shows the absorption spectra recorded for the HQ-containing ethanol solution (HQ/EtOH) and APA sample (v) immersed in this solution (HQ/APA). Interestingly, the absorption spectrum for HQ/APA does not show a peak at 313 nm due to the presence of HQ species, but exhibits a peak at 358 nm instead. The obtained absorption spectrum for HQ/APA matches well that for Alq_3_/APA, which is shown in Figure 4a. Although no fluorescence was observed from the HQ/EtOH solution, the fluorescence spectrum obtained for HQ/APA was very similar to that recorded for Alq_3_/APA (Figure 5b), indicating the formation of Alq_3_ from HQ species on the APA surface.

**Figure 6:**
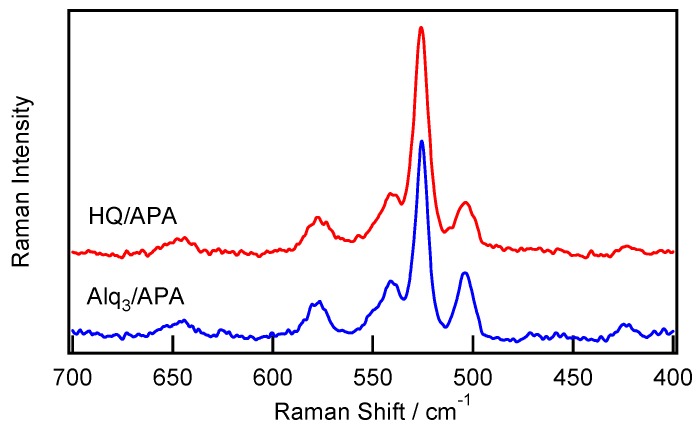
Raman spectra obtained for the HQ/APA (v) and Alq_3_/APA (v) samples in the 400–700 cm^−1^ region. The excitation wavelength was 785 nm.

Figure 6 shows the Raman spectra recorded in the 400–700 cm^−1^ region for the HQ/APA and Alq_3_/APA samples prepared under the conditions specified in Figure 4 and Figure 5. The main HQ/APA and Alq_3_/APA bands exhibit the same peaks at 423, 503, 525, 541, 577, and 645 cm**^−^**^1^, which are identical to those obtained in a previous study [27]. According to the assignments listed in [27], these Raman bands can be attributed to the following modes. The strongest band at 525 cm^−1^ is assigned to Al–O bond stretching and ring deformation of the quinoline species attached to Al atoms. The weak band at 541 cm^−1^ corresponds to ring deformation as well as to Al–O and Al–N stretching vibrations. The broad band at 577 cm^−1^ is assigned to ring deformation and Al–O–C bending. Therefore, the Raman spectrum for HQ/APA contains a direct evidence of the formation of Alq_3_ species from HQ on the APA surface. To avoid confusion, these species will be abbreviated as h-Alq_3_ further in the text.

### 2.3. Spectroscopic Characterization of Alq_3_- and h-Alq_3_-Species on the APA Samples Anodized under Various Conditions

**Figure 7:**
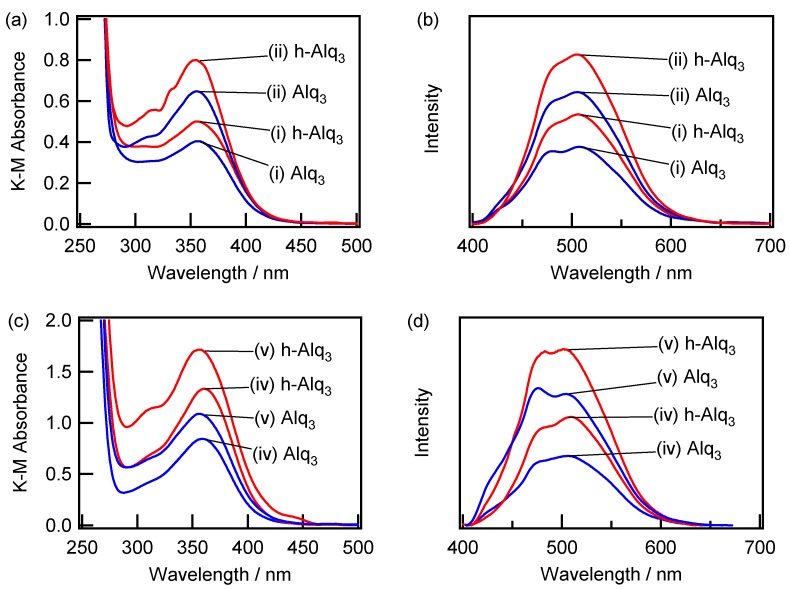
Absorption (**a**,**c**) and fluorescence (**b**,**d**) spectra recorded for the Alq_3_/APA and h-Alq_3_/APA samples prepared under various conditions. The excitation wavelength was 350 nm.

The corresponding absorption and fluorescence spectra obtained in this study exhibited similar features regardless of the preparation conditions, which indicate the adsorption of Alq_3_ and formation of h-Alq_3_ species on various surfaces ((i)–(vi)). Figure 7 shows the absorption (a) and fluorescence (b) spectra recorded for the Alq_3_/APA and h-Alq_3_/APA samples prepared under conditions (i) (via one-step anodization) and (ii) (via two-step anodization) at a constant potential, as well as the absorption (c) and fluorescence (d) spectra for the Alq_3_/APA and h-Alq_3_/APA systems prepared by one-step anodization at a constant current density with (iv) electropolishing and (v) degreasing pretreatment, respectively. The absorption spectra obtained for the Alq_3_/APA and h-Alq_3_/APA samples exhibit the same peaks at 355–358 nm. However, the K–M absorbance varied depending on the preparation conditions in the following order: (i) < (ii) < (iv) < (v), (see also the discussion in Section 2.4). The related fluorescence spectra (Figure 7b,d) also show two broad peaks centered at around 480 and 505 nm, which are similar to those depicted in Figure 5b.

### 2.4. Adsorption and Desorption Characteristics of Alq_3_ and h-Alq_3_ Species on Various APA Surfaces

**Figure 8:**
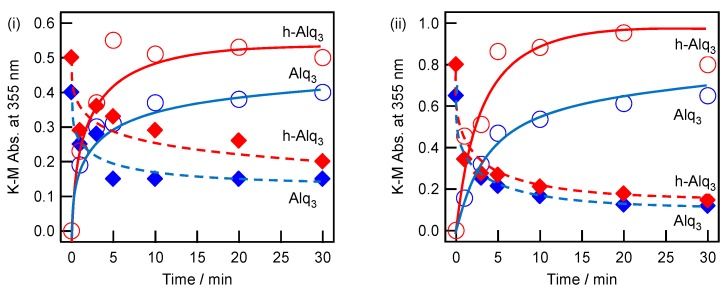
Time-dependence of the K–M absorbance at 355 nm for the Alq_3_/APA (○) and h-Alq_3_/APA (○) samples immersed in 1 mM Alq_3_/EtOH and HQ/EtOH solutions respectively, and for the samples of Alq_3_/APA (◆) and h-Alq_3_/APA (◆) in distilled water after 30-min adsorption. The notations (**i**,**ii**) correspond to the preparation conditions listed in Table 1.

The adsorption and desorption characteristics of Alq_3_ and h-Alq_3_ species on various APA surfaces were examined under the conditions listed in Table 1. Thus, Figure 8 shows the changes in the K–M absorbance observed for the Alq_3_ and h-Alq_3_ molecules on the APA (i) and (ii) surfaces at 355 nm during adsorption (blue and red lines) in 1 mM Alq_3_/EtOH or HQ/EtOH solutions and desorption in water (blue and red dashed lines) over time. Generally, the absorbance of Alq_3_ and h-Alq_3_ becomes virtually stable after about 10 min of reaction for both the adsorption and desorption processes. Table 3 and Table 4 summarize the peak absorbance values obtained after 30 min of adsorption (*Ads._30_*) and that 30 min of desorption (*Des._30_*) at a wavelength of 355 nm, which correlate with the remaining Alq_3_ amount, desorbed amount (*∆* = *Ads._30_* − *Des._30_*), desorption fraction (*Dr*% = *∆*/*Ads._30_* × 100), and the *Ads._30_* and *Des._30_* ratios of h-Alq_3_ to Alq_3_ for various APA surfaces, respectively.

**Figure 9:**
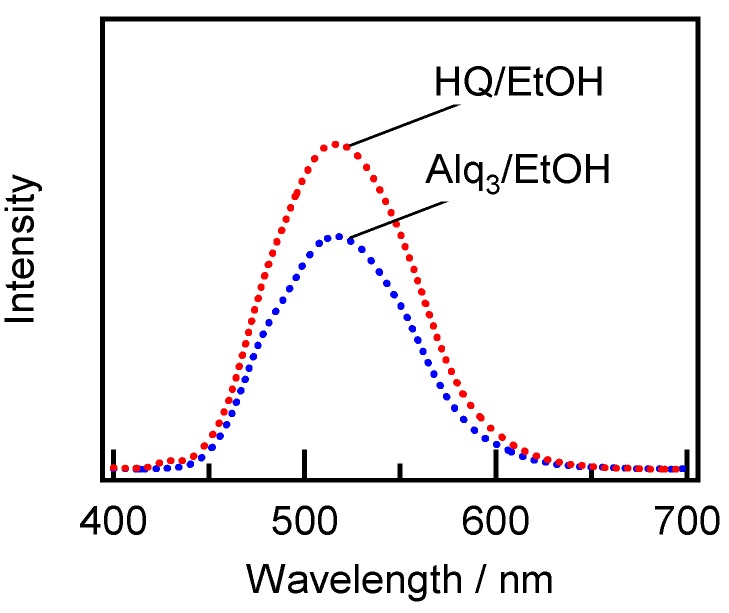
Fluorescence spectra obtained for the residual solutions of 1 mM Alq_3_/EtOH and HQ/EtOH after dipping and removal of the APA plates (v). The excitation wavelength was 380 nm.

Figure 9 depicts the fluorescence spectra for the residual solutions of Alq_3_/EtOH and HQ/EtOH after dipping and removal of the APA plates, which show a good agreement with each other. As mentioned earlier, the HQ/EtOH solution did not exhibit any fluorescence before APA immersion. Hence, the results indicate that the existence of h-Alq_3_ species is observed in the HQ/EtOH solution as well as the adsorbed h-Alq_3_/APA. Using the obtained data, a possible formation mechanism of Alq_3_ from HQ in solution can be proposed in addition to the adsorption and desorption results discussed earlier.

## 3. Discussion

### 3.1. Adsorption and Desorption of Alq_3_ and h-Alq_3_ Species 

**Figure 10:**
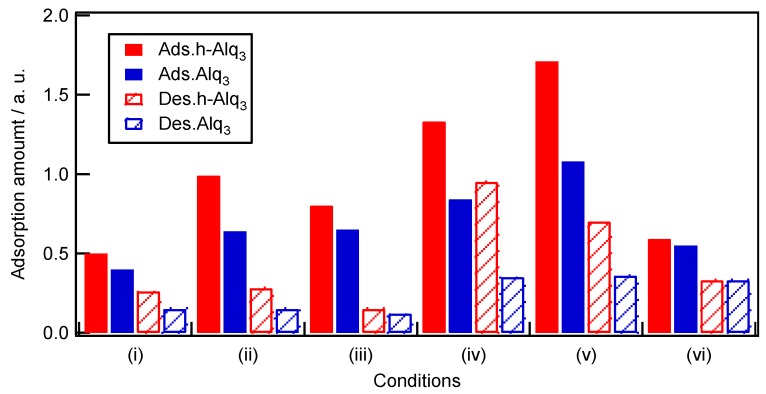
Relative adsorbed amounts of Alq_3_ and h-Alq_3_ on various APA surfaces obtained after immersion in 1 mM Alq_3_/EtOH and HQ/EtOH solutions for 30 min, respectively, and after treatment with water for 30 min.

Assuming that the adsorbed amount is proportional to the K–M absorbance, the latter parameter can be used as a measure of the Alq_3_ and h-Alq_3_ species adsorbed on the APA surface. Figure 10 displays the magnitudes of *Ads._30_* and *Des._30_* obtained for the Alq_3_ and h-Alq_3_ species (see Table 3).

In general, the adsorption amount of h-Alq_3_ was 1.1–1.6 times larger than that of Alq_3_ under all studied conditions (Table 4), and its highest magnitude was obtained for the APA (v) surface. First, the adsorption of Alq_3_ species must be discussed. Among the results obtained under potentiostatic conditions (i), (ii), and (iii), the adsorbed amounts on the APA (ii) and (iii) surfaces (produced via two-step anodization) were about 1.6–2.0 times larger than that on the APA (i) surface fabricated via one-step anodization. However, no significant correlation was found between the adsorption amount and APA structure parameters listed in Table 2: for example, *D_p_* of (i) 47 nm, (ii) 33 nm, and (iii) 18 nm; thickness of (i) 12μm, (ii) 14 μm, and (iii) 10 μm. The reason for this phenomenon can be a possible pore disordering, since Alq_3_/EtOH can more easily penetrate and adsorb on the surface of the ordered pores than the disordered ones. On the contrary, the residual amounts were very similar values (the average value of 0.14).

Next, the results obtained under galvanostatic conditions (iv, v, and vi) will be considered. The measured adsorption amounts can be ranked in the following order: (vi) << (iv) < (v). Unfortunately, it is difficult to compare the related APA structure parameters for these samples because of the insufficient number of available pore characteristics (Table 2). However, the observed pore structure effect was not significantly different from the results obtained under the potentiostatic conditions discussed above. A comparison between samples (v) and (vi) produced by the same degreasing pretreatment procedure indicates that the adsorption amount of Alq_3_ increases with an increase in the concentration of sulfuric acid electrolyte, because the number of species attached to the APA surface (v) (prepared in the 1.5 M sulfuric acid solution) was about twice as large as that on the surface fabricated in the 0.3 M sulfuric acid (vi). In addition, the selected surface pretreatment procedure appears to produce some effect on the adsorption process because the adsorbed amount on the degreased APA surface (v) was 1.3 times larger than that on the electropolished one (iv). Interestingly, the Alq_3_ amount remained after desorption exhibited almost the same value of about 0.35 for the (iv), (v), and (vi) surfaces regardless of the corresponding adsorbed quantities. The phenomenon is similar to that observed under the potentiostatic conditions, suggesting the existence of at least two Alq_3_ adsorption sites; one site can adsorb and desorb Alq_3_ easily and another one can strongly adsorb Alq_3_. These amounts depend on the potentiostatic and galvanostatic conditions. 

It is difficult to compare the effect of potentiostatic and galvanostatic anodizing directly, because the obtained samples were not suitable for evaluating the anodizing effect alone. However, the values of *Ads._30_* obtained for the (i) and (vi) samples, which were prepared under almost identical conditions, except for the electropolishing and degreasing, were very close to each other (Table 3). The most striking difference was clearly observed for the related amounts of *Des._30_* (0.33 for sample (vi) and 0.15 for sample (i)). On average, the *Des._30_* values obtained after galvanostatic anodization were about 2.5 times larger than those measured after potentiostatic anodization, indicating that the former method was capable of producing adsorption sites with higher binding strengths. In general, the pore diameter *D_p_* is linearly proportional to the anodizing potential *U* [1]:

*D_p_* = λ_p_ × *U* (3)

where λ_p_ is the proportionality constant (approximately 1.29 nm/V). During galvanostatic anodization, the potential increases linearly with time until a local maximum is reached due to the growth of a highly resistant barrier layer, and then gradually decreases to a constant value after the breakdown of the barrier layer caused by the growth of a porous structure [1,3]. Thus, the observed variations of *D_p_* can be caused by the change in potential, resulting in the growth of irregular pores (as compared to the ones produced during potentiostatic anodization). The pore irregularity and related surface roughness can be a reason for increasing the number of relatively stronger adsorption sites in contrast to the neat pore surface produced via potentiostatic anodization. 

The adsorption behavior of h-Alq_3_ species was very similar to that of Alq_3_ (see Table 3 and Figure 10), although the adsorbed amounts of h-Alq_3_ were 1.1–1.6 times larger than those of Alq_3_ under all studied conditions (Table 4). It is very likely that smaller HQ molecules can penetrate the pores more easily than Alq_3_ species, resulting in larger adsorbed amounts of h-Alq_3_. For the desorption process, the average desorption fractions *Dr*% for h-Alq_3_ and Alq_3_ species were equal to 56% and 65%, respectively (Table 3), corresponding to an increase in *Des._30_* ratio of h-Alq_3_ to Alq_3_ compared with *Ads._30_* of h-Alq_3_ to Alq_3_ (Table 4). The obtained results are consistent with the relatively stronger h-Alq_3_ adsorption sites as compared to those for Alq_3_ species discussed above.

### 3.2. Alq_3_ and h-Alq_3_ Adsorption Sites

In this section, we discuss the adsorption sites corresponding to the obtained spectroscopic data. For various APA samples, the related absorption spectra exhibited peak blueshifts from 377 nm (Alq_3_/EtOH) to around 360 nm (Alq_3_/APA). Remarkably, the fluorescence spectra exhibited two blueshifted peaks at around 480 and 505 nm from 513 nm. In general, the obtained results are consistent with the previously reported data except for the observed two peaks. Xu et al. [14,15] reported the absorption peak blue-shifted from 390 nm to around 360 nm for the APA samples immersed in a chloroform solution of Alq_3_. Fluorescence peak blueshifts were also observed for Alq_3_ on the APA surface, although the obtained results varied depending on the experimental conditions: (1) from 520 nm for the APA surface with pores of about 10–15 nm in diameter, which was dipped in a 10 mM chloroform solution of Alq_3_, to 488 nm for the same surface rinsed with chloroform (for the APA pores with diameters of 40–50 nm, the corresponding values were equal to 518 and 510 nm, respectively) [14,15]; (2) from 516 nm for a solid Alq_3_ film to 498 nm for Alq_3_-embedded APA, which was observed for the nanopore sizes of 23 and 45 nm after APA immersion in a 1 mg/mL 1,2-dichloroethane solution of Alq_3_ [9]; and (3) from 510 nm for APA dipped and dried several times in a 10 mM solution of Alq_3_ to 498 nm for the APA surface immersed for 21 h in a 1 mM chloroform solution of Alq_3_ and subsequently rinsed with chloroform [16]. Based on the obtained results, the fluorescence blueshift for Alq_3_ adsorbed on the APA pore surface could be explained by the suppression of Alq_3_ aggregation inside the APA nanopores [14,15] or nanovoids [9,16]. 

In addition to the investigation of nanopore-entrapped Alq_3_ species, various studies has been conducted for other porous systems such as porous glasses [28], sol-gel silica and zirconia materials [29], and mesoporous silica [30,31], which also reported blueshifts of the absorption and fluorescence peaks. In this study, we discuss the related results, containing two fluorescence peaks [30,31]. These papers described in detail the absorption and fluorescence of the Alq_3_ species adsorbed on the surface of mesoporous silica with different pores sizes (2.5, 3.1, and 5.0 nm) dipped in an absolute ethanol solution of Alq_3_ at room temperature for 1 h. The absorption bands obtained for Alq_3_ adsorbed on the mesoporous silica exhibited peak blueshifts from 255 and 375 nm (in EtOH) to 250 and 365 nm, respectively (regardless of the pore size). These blueshifts were attributed to a decrease in the Coulombic interaction energies between the Alq_3_ molecules confined in a nanospace, resulting in a wider bandgap value. In contrast to the absorption spectra, the obtained fluorescence spectra were more sensitive to the adsorbed amount of Alq_3_ and the pore size of mesoporous silica; two maxima were observed at around 470 and 520 nm, and the ratio between their intensities (*I*_520_*/I*_470_) gradually increased with an increase in the amount of Alq_3_ adsorbed inside the pores with sizes of 2.5 and 3.1 nm. Therefore, these two bands were attributed to isolated and aggregated Alq_3_ species inside the mesopores, respectively. In contrast, the fluorescence for the pores with sizes of 5.0 nm showed a single broad peak at 510 nm, indicating that the pore size of 5.0 nm was too large to isolate Alq_3_ species due to their significantly smaller molecular sizes (0.8 nm [9]–1.5 nm [31]).

**Figure 11:**
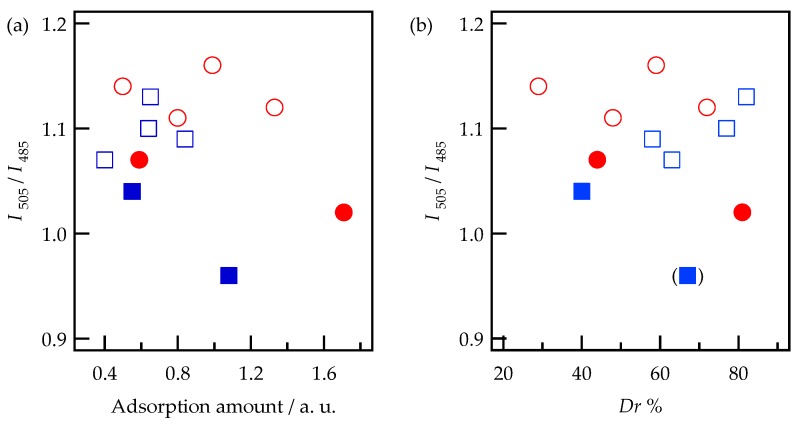
A ratio between the fluorescence peak intensities at 505 nm and 485 nm (*I*_505_/*I*_485_) plotted against (**a**) the relative adsorbed amount and (**b**) desorption fraction (*Dr*%) of Alq_3_ (□) and h-Alq_3_ (○) species on the APA surface (the unfilled and filled points correspond to electropolishing and degreasing pretreatment, respectively). The symbol (■) denotes the results obtained for the APA sample (v).

Form the comparison of the results obtained for APA and mesoporous silica, the present fluorescence peaks at 480 and 505 nm can be attributed to isolated Alq_3_ molecules inside the nanovoids and aggregated Alq_3_ clusters in the pores of APA, respectively. According to the analysis method described in references [30,31], the value of *I*_505_/*I*_480_ was plotted as a function of the relative adsorbed amount of Alq_3_ (Figure 11a). In contrast to the previous results obtained for mesoporous silica [30,31], no clear correlation between these two parameters was observed for APA. However, the value of *I*_505_/*I*_480_ apparently increased with an increase in the desorption fraction *D_r_*% except for sample (v), as shown in Figure 11b, which can be explained by the existence of two different adsorption sites suggested earlier. Namely, it can be considered that the isolated Alq_3_ molecules (characterized by the fluorescence peak at 480 nm) are more strongly adsorbed inside the nanovoids than the aggregated Alq_3_ clusters adsorbed on the pore surface (corresponding to the peak at 505 nm). As discussed in Section 3.1, Alq_3_ amounts remained after desorption were 0.14 and 0.35 for the APA prepared under the potentiostatic and galvanostatic conditions, respectively. If the above assignments are correct, it may be suggested that the galvanostatic conditions create more voids that the potentiostatic ones within our experiment. The exceptional behavior of sample (v) may be attributed to the surface covered with the cracked and pin-holed oxide layer and the APA porous structure (Figure 3), which increases the fraction of the isolated Alq_3_ molecules.

In contrast, no substantial correlations were detected for h-Alq_3_ species (as indicated in Figure 11a,b), which can be related to their formation and adsorption mechanism. Although additional data collection is required to elucidate it in more detail, the obtained *I*_505_*/I*_480_ values for the degreased APA samples (v) and (vi) were slightly smaller than those measured for the electropolished APA samples. Thus, both the rough surface morphology and APA porous structure may affect the h-Alq_3_ formation and adsorption processes. 

### 3.3. Reaction and Entrapment of HQ Species with APA Surface

**Figure 12:**
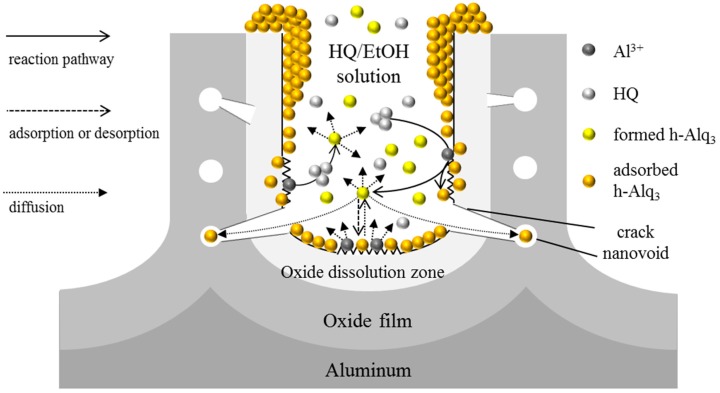
Formation of Alq_3_ species inside the APA nanopores.

As have been previously discussed in the Results section, h-Alq_3_ molecules are formed on the APA surface immersed in the HQ/EtOH solution (see Figure 5, Figure 6 and Figure 7), and their residual amounts remain in this solution after dipping and removing the APA plate (see Figure 9). According to the model proposed for the anodic process of alumina [1], the field-assisted dissolution of porous alumina occurs in the oxide dissolution zone located near the oxide/electrolyte interface, and the resulting Al^3+^ ions migrate towards the bulk electrolyte. Therefore, Al^3+^ ions are widely distributed in the oxide dissolution zone after anodization and can adsorb on the surface of immersed APA plates. In addition, there are significant number of nanovoids and cracks extending from the surface to inside oxide film. Using the APA adsorption model described above, which takes into account the formation of h-Alq_3_ species on the APA surface immersed in the HQ/EtOH solutions, the following two possible formation mechanisms of h-Alq_3_ can be proposed (Figure 12). According to the first mechanism, HQ molecules penetrate into the oxide dissolution zone near the interface through cracks and react with Al^3+^ ions, forming h-Alq_3_ species, some of which remain in this zone, while others dissolve in EtOH. The second mechanism suggests that the adsorbed Al^3+^ ions dissolve in EtOH and react with HQ molecules to form h-Alq_3_. In both cases, some Alq_3_ molecules remain in the EtOH solution, while others adsorb inside the nanovoids and on the APA surface (in accordance with the established adsorption-desorption equilibrium).

The adsorbed amounts of h-Alq_3_ obtained after 30 min of immersing in a 1 mM HQ/EtOH solution were about 1.1–1.6 times larger than the number of Alq_3_ species measured after immersing in a 1 mM Alq_3_/EtOH solution for the same time. Assuming the stoichiometric reaction of 1 mM HQ/EtOH with an excess amount of Al^3+^, the concentration of the produced h-Alq_3_ species should not exceed 1/3 mM, implying that the effective fraction of entrapped h-Alq_3_ molecules would be 3–5 times larger than that of Alq_3_. In such a case, HQ molecules are capable of penetrating the APA nanovoids and nanopores more easily than Alq_3_ species because of their smaller sizes, resulting in larger adsorbed amounts of h-Alq_3_. In addition, the existence of relatively stronger adsorption sites intrinsic to h-Alq_3_ was suggested (Section 3.1), which could be attributed to the formation of h-Alq_3_ species in the oxide dissolution zone. 

## 4. Materials and Methods

### 4.1. APA Preparation

The APA samples were prepared using aluminum plates (99.59% purity, Nilaco Co., Tokyo, Japan) under the conditions listed in Table 1. Two types of pretreatment were used before the anodizing stage: (1) electropolishing in a mixed solution of 60% perchloric acid and 99.5% ethanol (1:4 v/v) for about 2 min at a constant voltage of 10 V and room temperature (i–iv); and (2) washing in a 3 wt % weak alkaline degreaser (Top alclean 101, Okuno Chem. Ind. Co., Ltd., Osaka, Japan) aqueous solution at 60 °C for 5 min (v and vi).

Conditions (i)–(iii) corresponded to anodization in a 0.3 M sulfuric acid solution at a constant voltage. In particular, condition (i) was realized via one-step anodization performed for 60 min at a voltage of 20 V and room temperature. During two-step anodization, the first anodizing step was conducted in a 0.3 M sulfuric acid solution for 10 min at a constant voltage of (ii) 20 or (iii) 10 V, after which the prepared oxide film was removed by treatment in an aqueous solution containing 6.0 wt % of phosphoric acid and 1.8 wt % of chloric acid for 15 min at a temperature of around 60 °C. The second anodizing step was performed under the same conditions for (ii) 60 or (iii) 120 min.

Conditions (iv)–(vi) corresponded to anodization performed at a constant current density of 3 A/dm^2^. Samples (iv and v) and (vi) were prepared at room temperature via 30-min immersion in 1.5 M and 0.3 M sulfuric acid solutions, respectively.

### 4.2. Treatment of APA Samples with Alq_3_- and HQ-Containing EtOH Solutions

Alq_3_ and HQ (Both Tokyo Chemical Ind. Co., Ltd., Tokyo, Japan) reagents were used as-received without further purification. The prepared APA plates were immersed in a 1 mM Alq_3_/EtOH or HQ/EtOH solution at a temperature of about 60 °C for a specified period, removed from the solution, and dried in the vertical position in air for 2 min. The treated APA plates were subsequently characterized via diffuse reflectance spectroscopy. Afterwards, the same procedure was repeated at a total immersion time of 30 min. During desorption studies, all the described steps were performed in water.

### 4.3. APA Surface Characterization

SEM images of the APA surfaces were obtained with a scanning electron microscope Quanta 3D 200i (FEI Co., Hillsboro, OR, USA). Oxide film thicknesses of the prepared APA plates were measured via the eddy current method performed by using a Kett electric coating thickness tester LH-373 (Kett Co., Tokyo, Japan).

Absorption spectra for the solutions and diffuse reflectance spectra for the APA plates (which were transformed to absorption spectra by utilizing the Kubelka-Munk function) were recorded by using an ultraviolet-visible spectrophotometer V-570 (JASCO Co., Tokyo, Japan) with an integrating sphere unit ISN-470 (JASCO Co., Tokyo, Japan). Fluorescence spectra were obtained with a fluorescence spectrophotometer FP-6500 (JASCO Co., Tokyo, Japan) at an incidence angle of 23° to reduce the intensity of scattering light. Raman measurements were performed using a Raman spectrometer NRS-3200 (JASCO Co., Tokyo, Japan) equipped with a 785-nm laser. All spectroscopic measurements were conducted at room temperature.

## 5. Conclusions

The formation and entrapment of Alq_3_ molecules were successfully observed on the APA surface after immersion of APA plates in HQ-containing ethanol solutions, as confirmed by the obtained absorption, fluorescence, and Raman spectroscopy data. The fluorescence spectra exhibited two peaks at 480 and 505 nm, which could be possibly attributed to isolated Alq_3_ molecules in nanovoids and aggregated Alq_3_ species inside the APA nanopores, respectively. The following two mechanisms of the Alq_3_ formation were proposed. According to the first mechanism, HQ molecules penetrate the oxide dissolution zone through cracks in the pore surface and react with Al^3+^ ions, forming Alq_3_ species. The second proposed mechanism suggests that the Al^3+^ ions adsorbed on the pore surface first dissolve in EtOH and then react with HQ molecules to form Alq_3_ species. The adsorbed amounts of the Alq_3_ molecules produced from HQ were about 1.1–1.6 times higher than those adsorbed from the Alq_3_/EtOH solution, which could be attributed to the smaller sizes of HQ molecules (as compared to those of Alq_3_ species). The effects of the APA preparation conditions (galvanostatic or potentiostatic anodization, magnitudes of anodizing current and voltage, one-step or two-step anodizing, and concentration of sulfuric acid electrolyte) on the properties of the resulting Alq_3_-containing APA samples were examined as well. Among the utilized experimental parameters, the sulfuric acid concentration was the most important factor affecting the adsorption of Alq_3_ on the APA surface. The pore irregularity and surface roughness lead to the formation of relatively strong APA adsorption sites and nanovoids, which can possibly provide new insights into the entrapment of dyes and pigments on the APA surface. Furthermore, we anticipate that the described surface reactions of ligands with APA can be utilized in conventional aluminum dyeing techniques, various functional photochemical and photophysical materials, optical devices, and sensors.

## Figures and Tables

**Table 1:** Anodizing conditions utilized for preparation of APA surfaces in sulfuric acid either at a constant potential (potentiostatic method) or constant current (galvanostatic method) method via one-step or two-step anodization. The Al surfaces were pretreated by either electropolishing (EP) or alkaline degreasing (AD).

Anodization Method	#	Number of Steps	H_2_SO_4_ (M)	Potential (V)	Current Density (A/dm^2^)	Time (min)	Pre-Treatment
Potentio-static	i	1	0.3	20	ca. 1.5	60	EP
	ii	2	0.3	20	ca. 1.5	60	EP
	iii	2	0.3	10	ca. 0.4	120	EP
Galvano-static	iv	1	1.5	ca. 15	3	30	EP
	v	1	1.5	ca. 14	3	30	AD
	vi	1	0.3	ca. 25	3	30	AD

**Table 2:** Parameters of the APA samples prepared under the conditions listed in Table 1: *D_p_* (pore diameter), *D_c_* (cell diameter), *t* (oxide film thickness), *n* (pore density), and *S_p_* (inner pore surface area per 1 cm^2^ of APA surface).

#	*D_p_* (nm)	*D_c_* (nm)	*t* (μm)	*n* (10^10^ cm^−2^)	*S_p_* (10^16^ nm^2^·cm^−2^)
i	47	65	12	1.37	2.44
ii	33	52	14	2.14	3.11
iii	18	35	10	4.71	2.66
iv	41	75	16	1.03	2.12
v	20 *	35 *	15	4.71 *	4.44 *
vi	44 *	74 *	12	1.05 *	1.74 *

* Values obtained after electropolishing of the as-prepared surface.

**Table 3:** K–M absorbance for the Alq_3_/APA and h-Alq_3_/APA samples after 30-min adsorption (*Ads._30_*) in 1 mM Alq_3_/EtOH and HQ/EtOH solutions, respectively, and 30-min desorption (*Des._30_*) in water, as well as the corresponding desorbed amounts (*∆* = *Ads._30_* − *Des._30_*) and desorption fraction (*Dr%* = *∆*/*Ads._30_* × 100).

APA	K–M Absorbance at 355 nm			
	h-Alq_3_ *Ads._30_*:*Des._30_*	h-Alq_3_ *∆* (*Dr%*)	Alq_3_ *Ads._30_*:*Des._30_*	Alq_3_ *∆* (*Dr%*)
(i)	0.50:0.26	0.24 (48)	0.40:0.15	0.25 (63)
(ii)	0.99:0.28	0.71 (72)	0.64:0.15	0.49 (77)
(iii)	0.80:0.15	0.65 (81)	0.65:0.12	0.53 (82)
(iv)	1.33:0.95	0.38 (29)	0.84:0.35	0.49 (58)
(v)	1.71:0.70	1.01 (59)	1.08:0.36	0.72 (67)
(vi)	0.59:0.33	0.26 (44)	0.55:0.33	0.23 (40)

**Table 4:** *Ads._30_* and *Des._30_* ratios of h-Alq_3_ to Alq_3_ obtained for various APA surfaces.

APA	K–M Absorbance Ratio at 355 nm	
	*Ads._30_* Ratio of h-Alq_3_ to Alq_3_	*Des._30_* Ratio of h-Alq_3_ to Alq_3_
(i)	1.3	1.7
(ii)	1.5	1.9
(iii)	1.2	1.3
(iv)	1.6	2.7
(v)	1.6	1.9
(vi)	1.1	1.0

## Abbreviations

The following abbreviations are used in this manuscript:

APA	Anodic porous alumina
Alq_3_	Tris(8-hydqoxyquinolin)aluminum
HQ	8-Hydroxyquinoline
Alq_3_/EtOH	An ethanol solution containing Alq_3_
HQ/EtOH	An ethanol solution containing HQ
Alq_3_/APA	APA immersed in an Alq_3_/EtOH solution
HQ/APA	APA immersed in a HQ/EtOH solution
h-Alq_3_	Alq_3_ formed from HQ/APA

## References

[1] Sulka G.D., Eftekhari A. (2008). Nanostructured Materials in Electrochemistry.

[2] Diggle J.W., Downie T.C., Goulding C.W. (1966). Anodic oxide films on aluminum. Chem. Rev..

[3] Lee W., Park S.-J. (2014). Porous Anodic Aluminum Oxide: Anodization and Templated Synthesis of Functional Nanostructures. Chem. Rev..

[4] Masuda H., Fukuda K. (1995). Ordered Metal Nanohole Arrays Made by a Two-Step Replication of Honeycomb Structures of Anodic Alumina. Science.

[5] Oliveria C.P., Freitas R.G., Mattoso L.H.C., Pereira E.C., Eftekhari A. (2008). Nanostructured Materials in Electrochemistry.

[6] Santos A., Kumeria T., Losic D. (2014). Nanoporous Anodic Alumina: A Versatile Platform for Optical Biosensors. Materials.

[7] Kukhta A.V., Gorokh G.G., Kolesnik E.E., Mitkovets A.I., Taoubi M.I., Koshin Y.A., Mozalev A.M. (2002). Nanostructured alumina as a cathode of organic light-emitting device. Surf. Sci..

[8] Miura I., Okada Y., Kudoh S., Nakata M. (2004). Organic electroluminescence in porous alumina. Jpn. J. Appl. Phys..

[9] Huang G.S., Wu X.L., Xie Y., Kong F., Zhang Z.Y., Sie G.G., Chu O.K. (2005). Photoluminescence from 8-hydroxyquinoline aluminum embedded in porous anodic alumina membrane. Appl. Phys. Lett..

[10] Wang Z., Chen Z., Lan Z., Zhai X., Du W., Gong Q. (2007). Enhancement of Alq_3_ fluorescence by nanotextured silver films deposited on porous alumina substrates. Appl. Phys. Lett..

[11] Nuntawong N., Horprathum M., Eiamchai P., Wong-ek K., Patthansasettakul V., Chindaudom P. (2010). Surface-enhanced Raman scattering substrate of silver nanoparticles depositing on AAO template fabricated by magnetron sputtering. Vacuum.

[12] Das G., Patra N., Gopalakrishnan A., Proietti Zaccaria R., Toma A., Thorat S., Fabrizio E.D., Diaspro A., Salerno M. (2012). Fabrication of large-area ordered and reproducible nanostructures for SERS biosensor application. Analyst.

[13] Tang C.W., Vansyke S.A. (1987). Organic electroluminescent diodes. Appl. Phys. Lett..

[14] Xu C., Xue Q., Ba L., Zhao B., Gu N., Cui Y. (2001). Spectral behavior of 8-hydroxyquinoline aluminum in nanometer-sized holes of porous alumina. Chin. Sci. Bull..

[15] Xu C., Xue Q., Zhong Y., Cui Y., Ba L., Zhao B., Gu N. (2002). Photoluminescent blue-shift of organic molecules in nanometre pores. Nanotechnology.

[16] Mohammadpour A., Utkin I., Bodepudi S.C., Kar P., Fedosejevs R., Pramanik S., Shankar K. (2013). Photophysics and Energy Transfer Studies of Alq_3_ Confined in the Voids of Nanoporous Anodic Alumina. J. Nanosci. Nanotechnol..

[17] Ono S., Ichinose H., Masuko N. (1991). Defects in Porous Anodic Films Formed on High Purity Aluminum. J. Electrochem. Soc..

[18] Macdonald D.D. (1993). On the Formation of Voids in Anodic Oxide Films on Aluminum. J. Electrochem. Soc..

[19] Du Y., Cai W.L., Mo C.M., Chen J., Zhang L.D., Zhu X.G. (1999). Preparation and photoluminescence of alumina membranes with ordered pore arrays. Appl. Phys. Lett..

[20] Huang G.S., Wu X.L., Mei Y.F., Shao X.F., Siu G.G. (2003). Strong blue emission from anodic alumina membranes with ordered nanopore array. J. Appl. Phys..

[21] Santos A., Alba M., Rahman M.M., Formentín P., Ferré-Borrull J., Pallarés J., Marsal L.F. (2012). Structural tuning of photoluminescence in nanoporous anodic alumina by hard anodization in oxalic and malonic acids. Nanoscale Res. Lett..

[22] Yamaguchi S., Hidaka Y., Matsui K. (2015). Dyeing of Anodic Porous Alumina Using a Micellar Solution of Sodium Dodecyl Sulfate. Trans. Mater. Res. Soc. Jpn..

[23] Yamaguchi S., Matsui K. Synthesis of Alq_3_ by the Reaction of 8-Quinolinol with Anodic Porous Alumina. Proceedings of the 7th International Symposium on Surface Science.

[24] Yamaguchi S., Matsui K. Absorption and Fluorescence Properties of 8-Hydroxyquinoline in Anodic Porous Alumina with Ordered Holes. Proceedings of the IEEE International Conference on Applied System Innovation.

[25] Sulka G.D., Stępniowsli W.J. (2009). Structural features of self-organized nanopore arrays formed by anodization of aluminum in oxalic acid at relatively high temperatures. Electrochem. Acta.

[26] Hoshi T., Kumagai K., Inoue K., Enomoto S., Nobe Y., Kobayashi M. (2008). Electronic absorption and emission spectra of Alq_3_ in solution with special attention to a delayed fluorescence. J. Lumin..

[27] Braun M., Gmeiner J., Tzolov M., Coelle M., Meyer F.D., Milius W., Hillebrecht H., Wendland O., von Schütz J.U., Brütting W. (2001). A new crystalline phase of the electroluminescent material tris(8-hydroxyquinoline)aluminum exhibiting blueshifted fluorescence. J. Chem. Phys..

[28] Antropova T.V., Baran J., Gavrilko T., Gnatyuk I., Morawska-Kowal T., Melnik V., Puchkovska G., Vorobjev V. (2005). Interface interactions and optical properties of novel photonic nanocomposites consisting of porous glasses doped with organic luminophore molecules. Opt. Appl..

[29] Baldacchini G., Chiacchiaretta P., Reisfeld R., Zignsky E. (2009). The origin of luminescence blueshifts in Alq_3_ composites. J. Lumin..

[30] Tagaya M., Ogawa M. (2006). Luminescence of Tris(8-quinolinato)aluminum(III) (Alq_3_) Adsorbed into Mesoporous Silica. Chem. Lett..

[31] Tagaya M., Ogawa M. (2008). Possible pore size effects on the state of tris(8-quinolinato)aluminum(III) (Alq_3_) adsorbed into mesoporous silicas and their temperature dependence. Phys. Chem. Chem. Phys..

